# Increased pathologic complete response are expected in HER2-positive and triple-negative locally advanced breast cancers

**DOI:** 10.1186/s40880-017-0208-5

**Published:** 2017-05-10

**Authors:** Kadri Altundag

**Affiliations:** MKA Breast Cancer Clinic, Tepe Prime, Cankaya, 06800 Ankara, Turkey

## To The Editor

I want to congratulate Dr. Tan and colleagues for their article entitled “Weekly taxane-anthracycline combination regimen versus tri-weekly anthracycline-based regimen for the treatment of locally advanced breast cancer: a randomized controlled trial” [1]. The pathologic complete response (pCR) rate was similar in the two arms (10.61% vs. 12.31%, *P* = 0.665). However, the authors did not stratify patients according to molecular subtypes such as luminal A and B, human epidermal growth factor receptor 2 (HER2)-positive and triple-negative breast cancers (TNBC). Rouzier et al. [2] reported that the patients in TNBC and HER2-positive subgroups had the highest rates of pCR (45% and 45%), whereas the patients with luminal tumors had a pCR rate of 6% after neoadjuvant chemotherapy. Since HER2-positive and TNBC subgroups of tumors are more sensitive to chemotherapy, pCR rates in these tumors are expected to be more than 20%–25%. Taken all together, the evaluation of pCR rates after chemotherapy in locally advanced breast cancer patients would be better evaluated according to molecular subtypes.

I confirm that I have read BioMed Central’s guidance on competing interests. I have no competing interests in the manuscript.

### References

1. Tan QW, Luo T, Zheng H, Tian TL, He P, Chen J, et al. Weekly taxane-anthracycline combination regimen versus tri-weekly anthracycline-based regimen for the treatment of locally advanced breast cancer: a randomized controlled trial. Chin J Cancer. 2017; 36(1):27.
2. Rouzier R, Perou CM, Symmans WF, Ibrahim N, Cristofanilli M, Anderson K, et al. Breast cancer molecular subtypes respond differently to preoperative chemotherapy. Clin Cancer Res. 2005;11(16):5678–85.

## The author and colleagues reply

We thank Dr. Altundag for the letter discussing our recent study [1]. As mentioned in the Discussion section of our article, hormone receptor (HR)-positive breast cancers are less sensitive to chemotherapy than HR-negative breast cancers. A subgroup analysis would have been meaningful for our study. However, as you may notice, some important information including human epidermal growth factor receptor 2 (HER2) status were missing. As locally advanced breast cancers are relatively rare in clinical practice, it would have taken us more than 3 years to enroll 293 eligible women. If we excluded the patients with missing information, the sample size for subgroup analyses would have been too small. In addition, limited by the rarity of locally advanced breast cancers as well, we did not plan for a subgroup analysis during sample size calculations. Retrospective subgroup analyses in a prospective trial would reduce the test power and against the principle of statistics.

The pathologic complete response (pCR) rate reported in our study was similar with some other studies [2, 3]. The majority of patients in the study were HR-positive (70%), with a HER2-positive rate of less than 50%, which was significanty lower than those reported by Rouzier et al. [4]. We believe these variations may account for the overall differences in chemotherapy responses.

Qiu-Wen Tan^1^, Ting Luo^2^, Hong Zheng^2^, Ting-Lun Tian^2^, Ping He^2^, Jie Chen^1^, He-Lin Zeng^2^, Qing Lv^1*^

^1^ Department of Breast Surgery, West China Hospital, Sichuan University, Chengdu, Sichuan 610041, P. R. China

^2^ Department of Medical Oncology, West China Hospital, Sichuan University, Chengdu, Sichuan, 610041, P. R. China

*Correspondence: lvqingwestchina@163.com

### References

1. Tan QW, Luo T, Zheng H, Tian TL, He P, Chen J, et al. Weekly taxane-anthracycline combination regimen versus tri-weekly anthracycline-based regimen for the treatment of locally advanced breast cancer: a randomized controlled trial. Chin J Cancer. 2017; 36(1):27.
2. de Matteis A, Nuzzo F, D’Aiuto G, Labonia V, Landi G, Rossi E, et al. Docetaxel plus epidoxorubicin as neoadjuvant treatment in patients with large operable or locally advanced carcinoma of the breast: a single-center, phase II study. Cancer. 2002;94(4):895–901.
3. Steger GG, Galid A, Gnant M, Mlineritsch B, Lang A, Tausch C, et al. Pathologic complete response with six compared with three cycles of neoadjuvant epirubicin plus docetaxel and granulocyte colony-stimulating factor in operable breast cancer: results of ABCSG-14. J Clin Oncol. 2007;25(15):2012–8.
4. Rouzier R, Perou CM, Symmans WF, Ibrahim N, Cristofanilli M, Anderson K, et al. Breast cancer molecular subtypes respond differently to preoperative chemotherapy. Clin Cancer Res. 2005;11(16):5678–85.

